# Heteromerization fingerprints between bradykinin B2 and thromboxane TP receptors in native cells

**DOI:** 10.1371/journal.pone.0216908

**Published:** 2019-05-14

**Authors:** Oula K. Dagher, Miran A. Jaffa, Aïda Habib, Fuad N. Ziyadeh, Ayad A. Jaffa

**Affiliations:** 1 Department of Biochemistry and Molecular Genetics, Faculty of Medicine, American University of Beirut, Beirut, Lebanon; 2 Department of Epidemiology and Population Health, Faculty of Health Sciences, American University of Beirut, Beirut, Lebanon; 3 INSERM-U1149, CNRS-ERL8252, Centre de Recherche sur l'Inflammation, Paris, France; 4 Sorbonne Paris Cité, Laboratoire d'Excellence Inflamex, Faculté de Médecine, Site Xavier Bichat, Université Paris Diderot, Paris, France; 5 Department of Internal Medicine, Faculty of Medicine, American University of Beirut, Beirut, Lebanon; 6 Department of Medicine, Medical University of South Carolina, Charleston, South Carolina, United States of America; Max Delbruck Centrum fur Molekulare Medizin Berlin Buch, GERMANY

## Abstract

Bradykinin (BK) and thromboxane-A_2_ (TX-A_2_) are two vasoactive mediators that modulate vascular tone and inflammation via binding to their cognate “class A” G-protein coupled receptors (GPCRs), BK-B2 receptors (B2R) and TX-prostanoid receptors (TP), respectively. Both BK and TX-A_2_ lead to ERK1/2-mediated vascular smooth muscle cell (VSMC) proliferation and/or hypertrophy. While each of B2R and TP could form functional dimers with various GPCRs, the likelihood that B2R-TP heteromerization could contribute to their co-regulation has never been investigated. The main objective of this study was to investigate the mode of B2R and TP interaction in VSMC, and its possible impact on downstream signaling. Our findings revealed synergistically activated ERK1/2 following co-stimulation of rat VSMC with a subthreshold dose of BK and effective doses of the TP stable agonist, IBOP, possibly involving biased agonist signaling. Single detection of each of B2R and TP in VSMC, using in-situ proximity ligation assay (PLA), provided evidence of the constitutive expression of nuclear and extranuclear B2R and TP. Moreover, inspection of B2R-TP PLA signals in VSMC revealed agonist-modulated nuclear and extranuclear proximity between B2R and TP, whose quantification varied substantially following single versus dual agonist stimulations. B2R-TP interaction was further verified by the findings of co-immunoprecipitation (co-IP) analysis of VSMC lysates. To our knowledge, this is the first study that provides evidence supporting the existence of B2R-TP heteromerization fingerprints in primary cultured VSMC.

## Introduction

G-protein coupled receptors (GPCRs) constitute the largest family of membrane receptors and represent the targets for more than one third of globally marketed drugs [1,2]. Among the five classes of GPCRs, “class A” is the largest and most extensively studied. While some “class A” GPCRs could exist as perfectly functional monomers, many have also been found to be functional as dimers or oligomers [3]. With the vast majority of heteromerization studies being performed on cells overexpressing tagged GPCRs, only few studies have provided evidence of GPCR heteromerization within native cells or tissues [4–7].

Bradykinin (BK) B2 receptors (B2R) and thromboxane (TX) prostanoid receptors (TP) belong to “class A” GPCRs that are located on the cell surface of vascular smooth muscle cells (VSMC) [8–10]. In VSMC, the vasoactive peptide, BK, binds with high affinity to its B2R [8,9], and, through coupling to Gα_q_, results in PLC-mediated intracellular calcium mobilization [11,12]. In parallel, the vasoactive actions of the prostaglandin, TX-A_2_, in VSMC are primarily mediated by the human TPα isoform, via coupling mainly to Gα_q_ and Gα_12/13_ [13–17]. No other isoforms have been identified so far for both murine and rat TP, which have sequence homology with human TPα [10,18]. Several studies have demonstrated that each of B2R and TP could form functional dimers with various other GPCRs [19,20].

Several mechanisms of GPCR-induced ERK1/2 activation have been demonstrated, including generation of ROS, transactivation of receptor tyrosine kinases such as epidermal growth factor receptor (EGFR), scaffolding of β-arrestin, and activation of PKA, PKC, or Src kinases [21–26]. We and others have previously demonstrated that BK-bound B2R could promote mitogenesis and vascular fibrosis in rat VSMC through activating ERK1/2 [27,28]. The early ERK1/2 mediated mitogenic effect of BK on VSMC involved PKC, Src kinase, Grb2 and focal adhesion kinase (P^125^FAK)-mediated pathways. Likewise, TX could activate ERK1/2 in VSMC leading to mitogenic and/or hypertrophic effects [29,30]. In human VSMC, TP-induced ERK1/2 activation involved both Gα_q_ and Gα_i_-proteins, Src kinase, and PKC, and was mediated by transactivation of matrix metalloproteinases and EGFR [26].

The only well-established interface so far between BK and TX-A_2_ is the ability of the former, once bound to B2R, to activate the arachidonic acid/prostaglandin pathway and subsequently lead to increased TXA_2_ production in several systems including airway smooth muscle cells [31]. Although much is known about how stimulation of either B2R or TP in VSMC could activate the ERK1/2 MAPK in isolation leading to enhanced proliferation and/or hypertrophy, respectively [28,30], to our knowledge, no previous work has been done addressing how ERK1/2 activity is regulated when those receptors are activated simultaneously. While each of B2R and TP could form functional dimers with various GPCRs, the likelihood that B2R-TP heteromerization could contribute to their co-regulation has never been investigated. Here, we focused our work on investigating the mode of interaction between B2R and TP in VSMC at the signaling level and in terms of receptor-receptor interactions. We first examined the downstream signaling crosstalk between B2R and TP, with particular interest in ERK1/2 phosphorylation in VSMC, and the type of cooperation that would exist upon combined stimulation with their agonists. We next utilized in situ proximity ligation assay (PLA) and co-immunoprecipitation (co-IP) protocols for the assessment of the proximity and likelihood of heteromerization between B2R and TP in native VSMC without the need of using fluorescently labeled ligands or receptors. Our findings support the existence of B2R-TP interaction in VSMC with distinct signaling modalities.

## Materials and methods

### Reagents

IBOP ([1S-[1*α*,2*α*(Z),3*β*(1E,3S),4*α*]]-7-[3-[3-hydroxy-4-(4-iodophenoxy)-1-butenyl]-7-oxabicyclo[2.2.1]hept-2-yl]-5-heptenoic acid), SQ29548 ([(1S)1α,2β(5Z)3α,4β]-7-[3-[2-(phenylamino)carbonyl-hydrazino-methyl]-7-oxabicyclo-[2.2.1]-hept-2-yl]-5-heptenoic acid), and Gö6983 were obtained from Cayman Chemical Co. (Ann Arbor, MI, USA). AG1478 was from Calbiochem (San Diego, CA, USA). Bradykinin B-3259 (Arg-Pro-Pro-Gly-Phe-Ser-Pro-Phe-Arg), Dimethyl pimelimidate dihydrochloride (DMP), and Duolink detection kit were purchased from Sigma-Aldrich (St. Louis, MO, USA).

### Primary rat aortic smooth muscle cells (RASMC) isolation and culture

All rats were sacrificed in accordance with an approved protocol of the Institutional Animal Care and Use Committee (IACUC) at the American University of Beirut, Beirut, Lebanon. 100–150 g male Sprague Dawley rats were sacrificed, and aortas were then extracted as previously described with some modifications [28]. A 2 cm-segment of aorta was cleaned from adventitia and fat, then incubated with collagenase A solution (Roche Diagnostic, Indianapolis, USA) in serum-free, 1g/L glucose-containing Dulbecco’s modified Eagle medium (DMEM) (Sigma-Aldrich) supplemented with 1% penicillin, 20 mM HEPES (pH 7), and 1% L-Glutamine for 1 hr at 37°C in a water bath. This was followed by scraping of the aorta and grinding into fine aortic rings using the forceps. Finally, rings of each aorta were kept in a T75-flask and RASMC were maintained in growth medium supplemented with 10% fetal bovine serum (FBS) (Sigma-Aldrich), 1% penicillin, 20 mM HEPES (pH 7), and 1% L-Glutamine and placed at 37°C in a humidified atmosphere of 95% air-5% CO2. Culture medium was replaced every two days and cells were passaged every 7–10 days. Cells were serum-starved upon reaching 90% confluence. Prior to each experiment, cells were seeded onto six-well plates and incubated for 48 hrs in serum-free growth medium supplemented with 0.1% bovine serum albumin (BSA), 1% penicillin, 20 mM HEPES (pH 7), and 1% L-Glutamine. Serum-free medium was replaced 2 hrs prior to stimulation with appropriate treatment combinations. Experiments were conducted on cells between passages two and six, inclusive.

### Detection of alpha smooth muscle actin (α-SMA) expression by immunofluorescence

RASMC, plated onto coverslips coated with 0.1 mg/mL poly-D-lysine, were fixed with 4% paraformaldehyde for 10 min at room temperature. Afterwards, cells were washed with phosphate buffered saline (PBS) pH7.4 containing 0.1% sodium azide, permeabilized for 30 min with 0.5% Triton-X-100 in PBS, and blocked with PBS containing 0.1% sodium azide and 3% normal goat serum (NGS) for 30 min. Cells were then incubated with mouse monoclonal antibody against α-SMA (1A4- ab7817; Abcam; dilution 1/100) diluted in PBS containing 0.1% sodium azide and 1% NGS overnight at +4°C in a humidity chamber. Subsequently, cells were washed twice and incubated with goat anti-mouse AlexaFluor 568-conjugated secondary antibody (A11031, Thermo Fisher Scientific; dilution 1/500) diluted in PBS containing 0.1% sodium azide and 1% NGS for 1hr at room temperature. Afterwards, cells were washed twice and counterstained with DAPI. Washes were repeated twice, and coverslips were then inverted onto glass slides and mounted using Prolong Diamond antifade mountant (Thermo Fisher Scientific). Cells in the negative control were probed with goat anti-mouse AlexaFluor 568-conjugated secondary antibody in the absence of anti-α-SMA antibody. Confocal microscopy was performed using a 40xoil immersion objective of Zeiss LSM 710 scanning confocal microscope.

### Western blotting

RASMCs were plated on six-well plates (150,000 cells /well) and allowed to grow in 10% FBS-containing media (1g/L glucose DMEM). Subconfluent cells were serum-deprived for 48 hrs and starvation medium (containing 0.1% BSA) was changed 2 hrs prior to stimulation with agonists for 10 min. After incubation with the proper treatments, cells were washed with ice-cold PBS containing Ca^2+^ and Mg^2+^, pH 7.4, and lysed by incubating in ice-cold lysis buffer [25 mM Tris, pH 7.4, containing 1% (v/v) NP40, 150 mM NaCl, 1 mM EDTA, 5% (v/v) Glycerol, 1mM sodium pyrophosphate, 10 mM sodium fluoride, 2 mM sodium orthovanadate,1mM PMSF, 2μg/ml leupeptin, 2μg/ml aprotinin, and 1 mM Benzamidine] for 10 min. Lysates were then centrifuged at 15000 G for 15 min at +4°C. Clear supernatants were collected for protein quantification (Bradford assay) and western blotting. Protein samples were prepared in 4x Laemmli sample buffer, then separated by 10% SDS-PAGE gel and transferred onto supported nitrocellulose membranes (pore size of 0.45 μm). Blots were then blocked for at least 1 hr in Tris-buffered saline containing Tween 20 (TBS-T) [50mM Tris, pH 7.5, 250 mM NaCl and 0.1% (v/v) Tween 20], containing 5% (w/v) fat free milk powder. Blocked membranes were subsequently incubated overnight at 4°C with anti-phospho- ERK1/2 (Cell Signaling Technology; 4370S - dilution: 1:8000). Membranes were then washed, followed by incubation for 1hr at room temperature with peroxidase AffiniPure anti-rabbit secondary antibody (Jackson ImmunoResearch—dilution 1/6000). Washes were repeated, and bands finally revealed by BioRad Clarity Western enzymatic chemiluminescence (ECL) blotting substrates according to the manufacturer’s protocol. Membranes were then stripped with a mild stripping buffer, followed by reblotting overnight at +4°C with anti-total ERK2 (C-14) (Santa Cruz, sc-154—dilution: 1:6000) followed by washes and incubation for 1hr with peroxidase anti-rabbit secondary antibody (dilution 1/6000). Autoradiographs were scanned with Epson scanner. Band signals were quantified by densitometry using ImageJ^®^ and plotted as “fold/basal” phosphorylation using GraphPad Prism 6. Where necessary, experimental data were fitted to an EC_50_ model and plotted using GraFit 7 Erithacus software.

### Analysis of synergy between BK and IBOP

The ‘‘Fixed Concentration” method was used for combination analysis of BK and IBOP on the ERK1/2 pathway in RASMCs. Cells were simultaneously stimulated with a fixed minimally effective dose of BK (10^−11^ M) plus IBOP (in a serial dilution of 10^−11^ M -10^−7^ M) for 10 min. Experimental data were fitted to a four-parametric non-linear regression EC_50_ model (**Eq 1**) and plotted using GraFit 7 Erithacus software, where (s) represents the slope value.

$$E=\frac{Range}{1+{\left(\frac{EC_{50}}{C}\right)}^{s}}+\mathrm{Background}$$ (1)

The non-linear regression trendlines generated by GraFit provided a better fit for the actual data and were used to calculate the concentrations of agonists (as single agents or in combination) at a specific effect level for the subsequent calculations of combination index (CI) values based on Loewe’s model of additivity [32]. The CI method introduced by Chou and colleagues was utilized in our work to examine the nature of BK-IBOP interactions by evaluating CI values at distinct effect levels (Fa, fraction affected; fold increase in ERK1/2 phosphorylation) to determine whether the interactions were antagonistic (if CI > 1), additive (if CI = 1), or synergistic (if CI < 1) [33–35]

CI values derived from non-linear regression curves were calculated using **Eq 2** in which *D*_*A*_ and *D*_*B*_ are the concentrations of BK and IBOP, respectively, in the combination, to produce effect (*x)* (fold increase in ERK1/2 phosphorylation). *D*_*x*,*A*_ and *D*_*x*,*B*_ are the concentrations of BK and IBOP, respectively, used as a single agent to produce that same effect (*x)*. *D*_*A*_, *D*_*B*_, *D*_*x*,*A*_ and *D*_*x*,*B*_ were calculated from **Eq 1** (stated above).

$$CI=\frac{D_{A}}{D_{x,A}}+\frac{D_{B}}{D_{x,B}}$$ (2)

CI values were calculated at five different effect levels reached with single agents (BK or IBOP) alone or in combination (fold/basal ERK1/2 phosphorylation: 2-fold, 3.5-fold, 4-fold, 5-fold, and 6-fold). Because our dose-response curves corresponding to BK alone and IBOP alone had different maxima (Emax) with E_max (IBOP)_ > E_max (BK)_, the effect levels were displayed as fraction affected (Fa) in the CI-Fa plot by normalizing to E_max (IBOP)_, as per **Eq 3**:

$$F=\frac{E_{i}}{E_{max(IBOP\;alone)}}$$ (3)

Dose-Reduction Index (DRI) values for BK and IBOP were calculated as per **Eq 4** then plotted in the form of Log(DRI)-Fa plot. A DRI > 1 (or Log(DRI) > 0) is favorable to allow for dose reduction for single agonists when used in combination to achieve a certain effect [33,35].

$$\mathrm{CI}=\frac{1}{{\mathrm{DRI}}_{\left(A\right)}}+\frac{1}{{\mathrm{DRI}}_{\left(B\right)}}$$ (4)

The normalized isobologram for the non-constant ratio combination design was also constructed for BK-IBOP combination with D_BK_/D_x,BK_ as the x-axis and D_IBOP_/D_x,IBOP_ as the y-axis [35].

### In-situ PLA

In-situ PLA was performed using Duolink detection kit as per manufacturer’s instructions (Sigma-Aldrich) for analysis of dual PLA receptor recognition (B2R-TP proximity), or single PLA receptor recognition of either B2R or TP in RASMC [36] (S2A Fig). Briefly, RASMC, seeded on poly-D-lysine coated, 8-well Permanox Nunc™ Lab-Tek chamber slides (Thermo Fisher Scientific), were starved for 48 hrs using FBS-free DMEM media containing 0.1% BSA. Cells were then kept untreated or stimulated with BK, IBOP, or combination thereof for 10 min. Cells were fixed with 4% paraformaldehyde, permeabilized with 0.5% Triton X-100, and blocked with Duolink blocking reagent for 1hr in a 37°C humidity chamber. Thereafter, the cells were incubated with primary antibodies followed by incubation with Duolink PLA MINUS and PLA PLUS proximity probes as detailed in S3 Table. Proximity ligation and detection were performed using the Duolink detection reagent kit as per the manufacturer’s instructions. The resulting amplified PLA signal was detected using hybridization probes labeled with Alexa 594. Duolink II Mounting Medium with DAPI was used for nuclear counterstaining. The term ‘PLA blob’ was used throughout this work to refer to the amplified PLA signal from each detected pair of PLA probes [36]. Confocal microscopy was performed using a 40xoil immersion objective of Zeiss LSM 710 scanning confocal microscope. Approximately five z-stacks from non-overlapping regions with an optimal interval distance between slices were captured per sample and repeated over three independent experiments. For each independent experiment, z-stacks were taken with the same acquisition parameters under the same imaging session.

PLA blobs were quantified by the BlobFinder_V3.2 freeware (http://www.cb.uu.se/~amin/BlobFinder) using the single cell analysis feature for subsequent data analysis. First, z-stacks were processed with Zen 3.2 lite software whereby a batch of files was created for each z-slice in a z-stack image with split channels, one representing nuclear stain, and the other representing the PLA stain (594 nm). This batch of z-slices for each image was subsequently imported into the Blobfinder, and the image processing configurations were adjusted for single cell analysis. The software creates borders around the nuclei and cytoplasm in each image, allowing the quantification and discrimination between nuclear and extra-nuclear blobs for each cell within the z-stacks.

### Co-IP

Crosslinking of Immunoglobulins (IgG) to Protein-A conjugated magnetic beads (Dynabeads protein-A; Thermo Fisher Scientific) was carried out prior to proceeding with immunoprecipitation of target antigens. The beads were first incubated with anti-B2R antibody (rabbit IgG; Thermo Fisher Scientific; 720288) prior to crosslinking. Crosslinking was performed by incubating the Dynabeads-protein-A-IgG complexes with 0.2 M triethanolamine pH 8.2 as crosslinking buffer, then with 20 mM of the chemical crosslinker, DMP, prepared in crosslinking buffer. The reaction was stopped by incubating the bead complexes with 0.1 M ethanolamine, pH 8.2 as blocking buffer. The beads were then washed and the trace unbound antibody that was not crosslinked with DMP was eluted using 0.5 M glycine-HCl pH 2.8 as mild elution buffer. Meanwhile, cells were harvested and lysed by ice-cold NP40 lysis buffer [25 mM Tris, pH 7.4, containing 1% (v/v) NP40, 150 mM NaCl, 1 mM EDTA, 5% (v/v) Glycerol, 1mM sodium pyrophosphate, 10 mM sodium fluoride, 2 mM sodium orthovanadate,1mM PMSF, 2μg/ml leupeptin, 2μg/ml aprotinin, and 1 mM Benzamidine] followed by quantification by Bradford assay. Dynabeads-protein-A-IgG were then incubated with protein lysates containing the target antigen (Ag). Elution was performed by mixing the Dynabeads-IgG-Ag complexes with urea-CHAPS buffer (8 M urea, 4% CHAPS, 130 mM DTT) plus 4x Laemmli sample buffer for 5 min, separation from Dynabeads, adjusting the pH to 7.4, and heating for 10 min at 70°C. The obtained eluates (E), along with their matching total cell lysates (L), were run onto a 10% SDS-PAGE gel. The obtained membranes were then immunoblotted with rabbit anti-TP antibody; dilution 1/2000 (validated and prepared by Habib A., Bolla M., and Creminon C.; unpublished data), stripped, and reblotted with anti-B2R antibody; dilution 1/750. Western blot analysis of membranes was carried out as described above and the developed signal was captured with Chemidoc (BioRad; Hercules, CA, USA).

### Data presentation and statistical analysis

GraphPad prism 6 was used for graphical presentation of the data. ERK1/2 Dose Response curves were fitted and plotted using GraFit 7 Erithacus software. Servier medical arts was used for schematic presentation of some figures. Statistical analysis was performed using SigmaStat software. All statistically analyzed data were expressed as mean ± SEM of at least three independent experiments. Normality test based on the Kolmogorov-Smirnov method was initially performed. Comparison between single B2R and TP expression by PLA was done using Mann-Whitney Rank Sum t-test after the normality test had failed. Compared datasets of more than two treatment conditions that passed the normality test were analyzed using One Way Analysis Of Variance (ANOVA) followed by appropriate post *hoc* analysis method for pairwise comparison analysis between the treatment groups. In cases where normality test had failed, Kruskal-Wallis One Way ANOVA on Ranks was applied followed by appropriate post *hoc* analysis method for pairwise comparison between the treatment groups. Two Way ANOVA was used followed by Holm-Sidak procedure as post *hoc* analysis for comparing data between two different dose response curves for ERK1/2 profiling. Differences in compared datasets of p-value < 0.05 were considered as statistically significant.

## Results

### Synergistic cooperation between B2R and TP on the ERK1/2 pathway in VSMC

**Fig 1:**
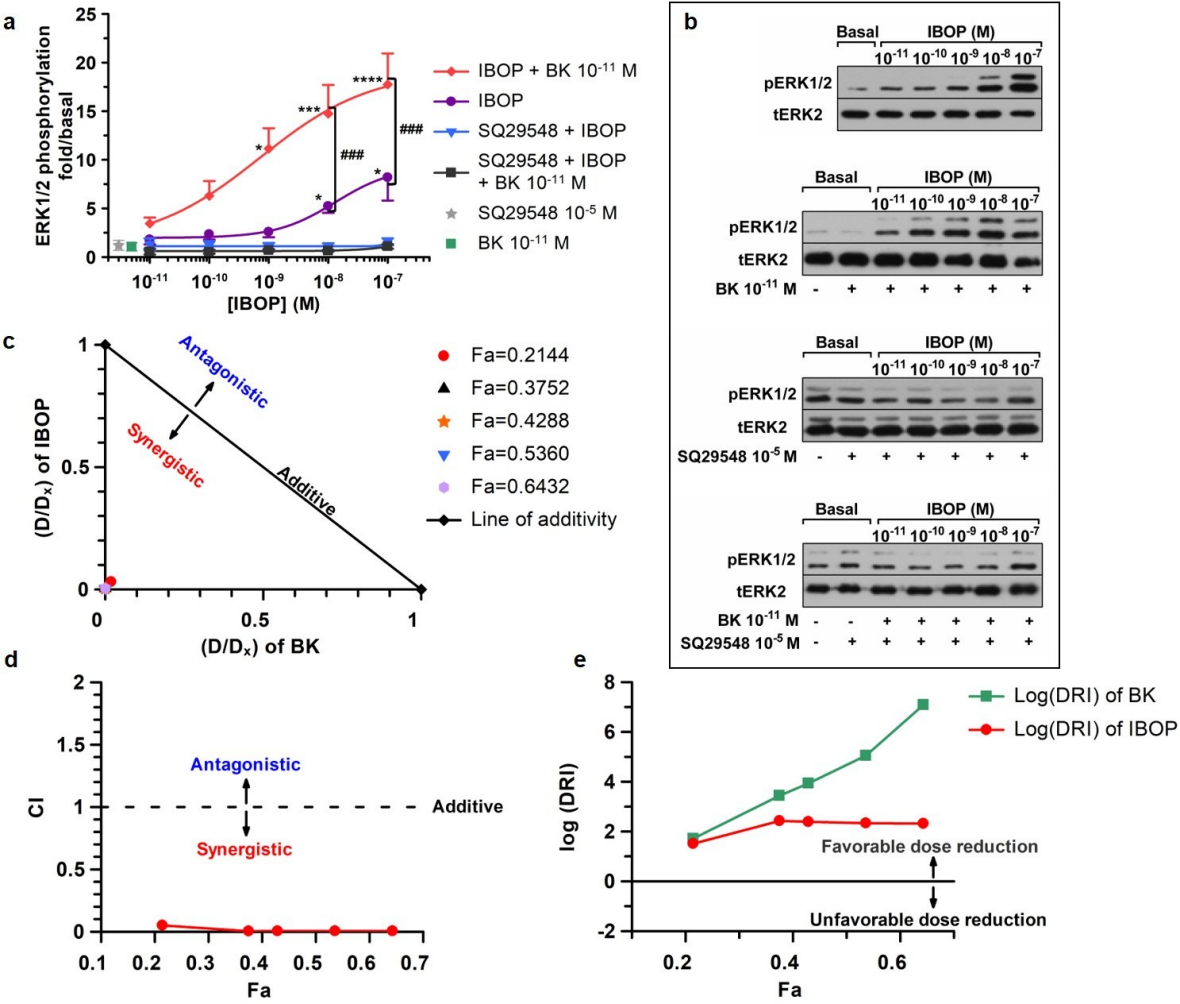
Synergistic ERK1/2 activation in (BK 10^−11^ M plus IBOP) co-stimulated RASMC. (a): Concentration-response curves representing “fold/basal” ERK1/2 phosphorylation in RASMC treated with IBOP alone or (BK 10^−11^ M plus IBOP) with or without prior incubation with the TP antagonist, SQ29548. Results are mean ± SEM of at least three independent experiments. * p < 0.05; ** p < 0.01; and *** p < 0.001 as compared to unstimulated basal, One Way ANOVA. (b): Western blots corresponding to curves in (a). (c, d and, e): Analysis of synergy in (BK 10^−11^ M and IBOP) binary combinations in RASMCs based on the method of Chou and colleagues at five different fractional effects (Fa). (c) The normalized isobologram analysis: The x-coordinate for each data point at a given Fa was calculated by dividing the concentration (D_A_) of BK in the (BK+IBOP) combination from its corresponding single-agent (D_x,A_) value. The y-coordinate was calculated by dividing the concentration (D_B_) of IBOP in the (BK+IBOP) combination from its corresponding single-agent (D_x,B_) value. Data points below the line of additivity indicate synergy, whereas points above the line of additivity indicate antagonism. (d) Combination index (CI) analysis: CI values at any given Fa were derived from actual data points in the dose-response non-linear regression curves. Combinations are additive at CI = 1, synergistic at CI < 1, and antagonistic at CI > 1. (e) Dose Reduction Index (DRI) was calculated at the above Fa levels and plotted as Log(DRI)-Fa plot, where Log(DRI) > 0 would be favorable in case of synergy.

Stable TP agonists such as IBOP have been widely utilized in research to mimic the actions of the unstable TX-A_2_ in-vitro [37]. First, we tested whether BK could modulate IBOP-induced ERK1/2 phosphorylation in VSMC. RASMC positive for α-SMA were used (S1A Fig). RASMC were made quiescent by serum starvation for forty-eight hours prior to treatment with increasing concentrations of IBOP (10^−11^ to 10^−7^ M) for 10 min, or with a subthreshold concentration of BK (10^−11^ M) (S1C and S1D Fig) plus increasing concentrations of IBOP (10^−11^ to 10^−7^ M) for 10 min (Fig 1A).

Findings in Fig 1A revealed a significant increase in ERK1/2 phosphorylation when BK 10^−11^ M was concomitantly added to concentrations of IBOP greater than or equal to 10^−9^ M as compared to basal (S1 Table). In addition, a significant increase in IBOP–induced ERK1/2 was obtained when RASMC were co-stimulated with a fixed subthreshold concentration of BK (10^−11^ M) plus IBOP (10^−9^ M or greater) as compared to the data corresponding to equivalent IBOP concentrations in cells individually treated with IBOP alone (S1 Table).

Under these conditions, SQ29548, a TP selective antagonist, totally inhibited any increase in ERK1/2 phosphorylation, when RASMC were pretreated with SQ29548 10^−5^ M for 30 min prior to stimulation with IBOP alone, or co-stimulation with a minimal concentration of BK (10^−11^ M) and variable concentrations of IBOP (10^−11^–10^−7^ M). This resulted in the significant reversal of ERK1/2 phosphorylation back to the basal level at all the treatment combinations applied (Fig 1A and 1C). Comparison between the curve corresponding to treatment with (SQ29548 + BK 10^−11^ M + IBOP) and that representing treatment with (BK 10^−11^ M + IBOP) revealed a significant difference in the treatment effect (p < 0.0001) and concentration effect (p = 0.0006) applied; Two Way ANOVA, post *hoc* Holm-Sidak.

The above findings demonstrate that a fixed subthreshold concentration of BK (10^−11^ M) could enhance the potency of IBOP. Visual inspection of the dose-response curves shows about a 2-fold increase in the E_max_ (E_max (combination)_ = 19.2025 versus E_max (IBOP alone)_ = 9.3276 folds), and an approximately 18-fold decrease in the EC_50_ value (from EC_50 (IBOP)_ = 1.32368x10^-8^ M in the case of single IBOP treatment, to EC_50 (combination)_ = 7.23257x10^-10^ M once combined with BK 10^−11^ M).

Since the “Fixed Concentration” model of drug combination was utilized in our work, the normalized isobolograms were first constructed to assess whether the realized positive modulatory effect of BK 10^−11^ M on IBOP-induced ERK1/2 phosphorylation was additive or synergistic [33]. The type of interaction between BK 10^−11^ M and IBOP was analyzed at five different effect levels that were reached with single agents (BK or IBOP) alone or in combination (fold/basal ERK1/2 phosphorylation: 2-fold, 3.5-fold, 4-fold, 5-fold, and 6-fold). Because our dose-response curves corresponding to BK alone and IBOP alone had different maxima (E_max_) with E_max (IBOP)_ > E_max (BK)_, the effect levels were displayed as fraction affected (Fa) by normalizing to E_max_ of IBOP alone. The corresponding Fa values were 0.2144, 0.3752, 0.4288, 0.5360, and 0.6432, respectively. Inspection of the normalized isobolograms shows that all our binary combination data points at the given Fa levels were below the line of additivity signifying strong synergy.

We next calculated the CI values at the above-mentioned Fa levels. The CI-Fa plots indicate that the data points at all given Fa levels were below the line CI = 1, indicating synergistic interaction in all the combination concentrations used. S2 Table shows that the calculated CI values at all Fa levels were below 0.1, which depicts very strong synergism between BK 10^−11^ M and IBOP [35]. All these findings are suggestive of a synergistic cooperation between BK 10^−11^ M and IBOP on the ERK1/2 pathway in RASMC.

Finally, the DRI values were calculated at the above-mentioned Fa levels. Since the DRI values were very high, a Log(DRI)-Fa plot was constructed for better presentation of the data. Likewise, Log(DRI) values at all given Fa levels showed favorable dose reduction as they were all positive. However, although the larger DRI value is beneficial and indicates a greater dose reduction for a given effect, it does not always reflect synergism, as it could also occur in the case of additivity [35].

### Divergent mechanisms of IBOP versus (BK+IBOP)-induced ERK1/2 activation in RASMC

We next analyzed, mechanistically, the mode of ERK1/2 activation in single versus double stimulated RASMC. Several mechanisms of GPCR-induced ERK1/2 activation have been demonstrated, prominent among which are activation of PKC and transactivation of EGFR (reviewed in [38]).

**Fig 2:**
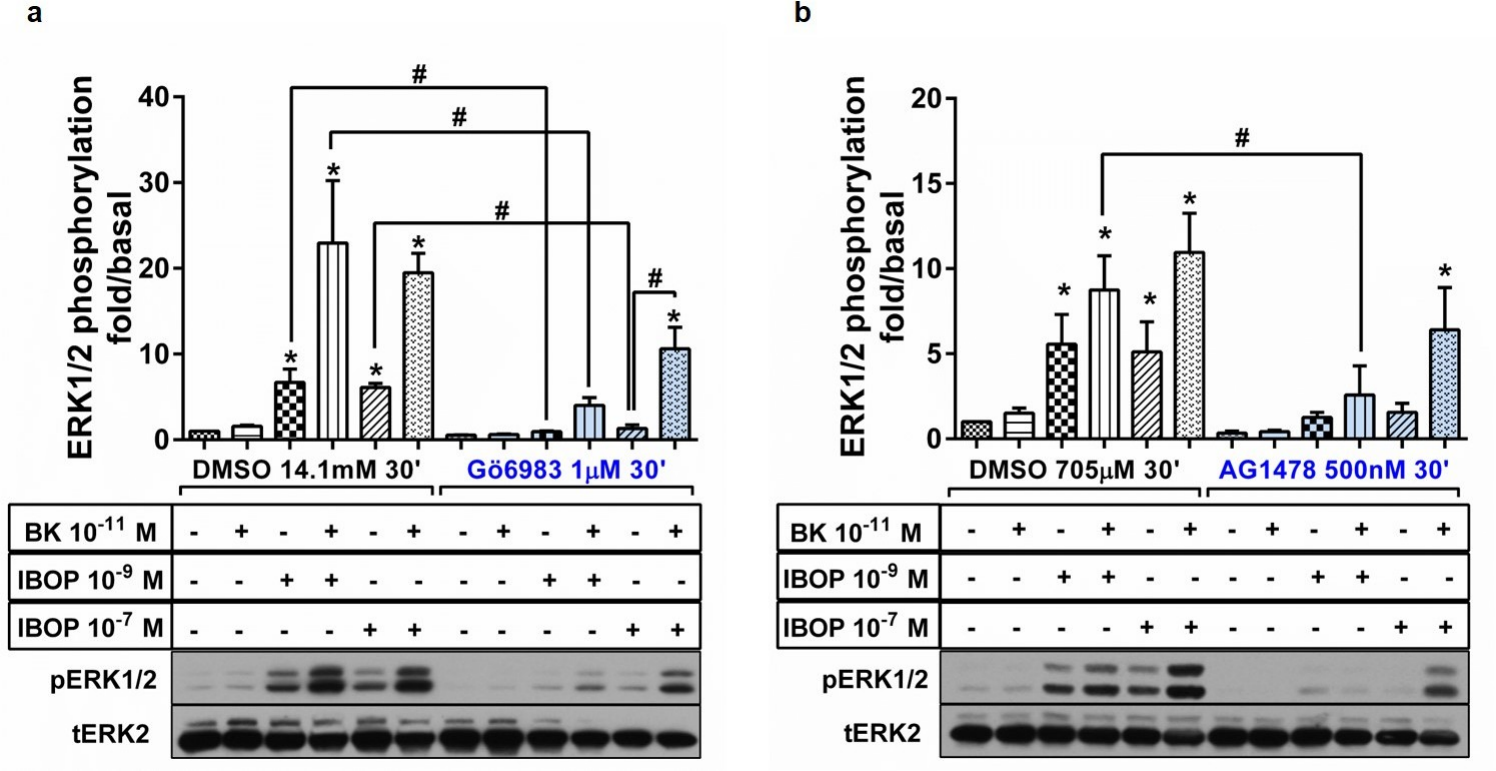
Differential mechanism of ERK1/2 activation in single versus double stimulation of B2R and TP in RASMC. Cells were pretreated with the PKC inhibitor, Gö6983 (a), or the EGFR Tyr kinase inhibitor, AG1478 (b), prior to stimulation with BK, IBOP, or combination. Results are plotted as “fold/basal” ERK1/2 phosphorylation. Representative western blots are seen for pERK1/2 and tERK2. Results are mean ± SEM of at least three independent experiments. *: p < 0.05; as compared to unstimulated basal. #: p < 0.05; for pairwise comparisons, One Way ANOVA.

Hence, we first examined the role of PKC in mediating IBOP versus (BK+IBOP)-induced ERK1/2 activation. RASMC were incubated with 1μM of the pan PKC inhibitor, Gö6983, for 30 min prior to stimulation with BK 10^−11^ M, IBOP 10^−9^ M or 10^−7^ M, or combinations thereof for 10 min. IBOP concentrations were chosen in the range of the EC_50_ concentration obtained in the IBOP dose-response curve. Notably, in IBOP-stimulated RASMC, PKC provided the predominant source of ERK1/2 activation, as seen with almost total reversal of ERK1/2 phosphorylation using Gö6983 (Fig 2A). This lends support to the previously established line of evidence of thromboxane-induced activation of the Gαq-PLC-PKC axis in VSMC [26]. However, the synergistic ERK1/2 activation in double stimulated cells was only partially inhibited following pretreatment with Gö6983. This suggests that another non-PKC mediated pathway is involved in (BK plus IBOP) synergistic ERK1/2 (Fig 2A).

Thus, we next assessed the role of EGFR transactivation in [BK + IBOP]-induced ERK1/2 synergy. RASMC were incubated with 500nM of AG1478, a highly selective inhibitor of intracellular tyrosine-dependent phosphorylation of EGFR, for 30 min prior to stimulation with BK 10^−11^ M, IBOP 10^−9^ M or 10^−7^ M, or combinations thereof for 10 min. Interestingly, AG1478 almost totally inhibited IBOP-induced ERK1/2 phosphorylation, irrespective of the concentration of IBOP applied (Fig 2B). However, distinct patterns of ERK1/2 attenuation were obtained for the (BK plus IBOP) double stimulated condition depending on IBOP concentration. While AG1478 promoted a significant reduction in ERK1/2 phosphorylation in (BK 10^−11^ M+ IBOP 10^−9^ M)-treated RASMC, ERK1/2 pathway retained several folds of activation in the (BK plus IBOP) double stimulated condition at the above EC_50_ dose of IBOP (10^−7^ M) (Fig 2B).

### Quantification of the subcellular prevalence of B2R, TP, and B2R-TP proximity in RASMC by in-situ PLA

#### a- Investigating the subcellular localization and distribution of B2R and TP in RASMC

Having established the signaling crosstalk downstream of B2R and TP, we next aimed to examine the proximity between B2R and TP and hence the likelihood of B2R-TP heteromerization in VSMC. We first assessed the single receptor occupancy of each of B2R and TP in unstimulated RASMC at the subcellular level, both quantitatively and qualitatively by utilizing the single receptor recognition workflow of in-situ PLA. This workflow would allow the concomitant detection and quantification of receptor monomers and/or homomers for each of B2R and TP that might exist in RASMC (S2A Fig).

**Fig 3:**
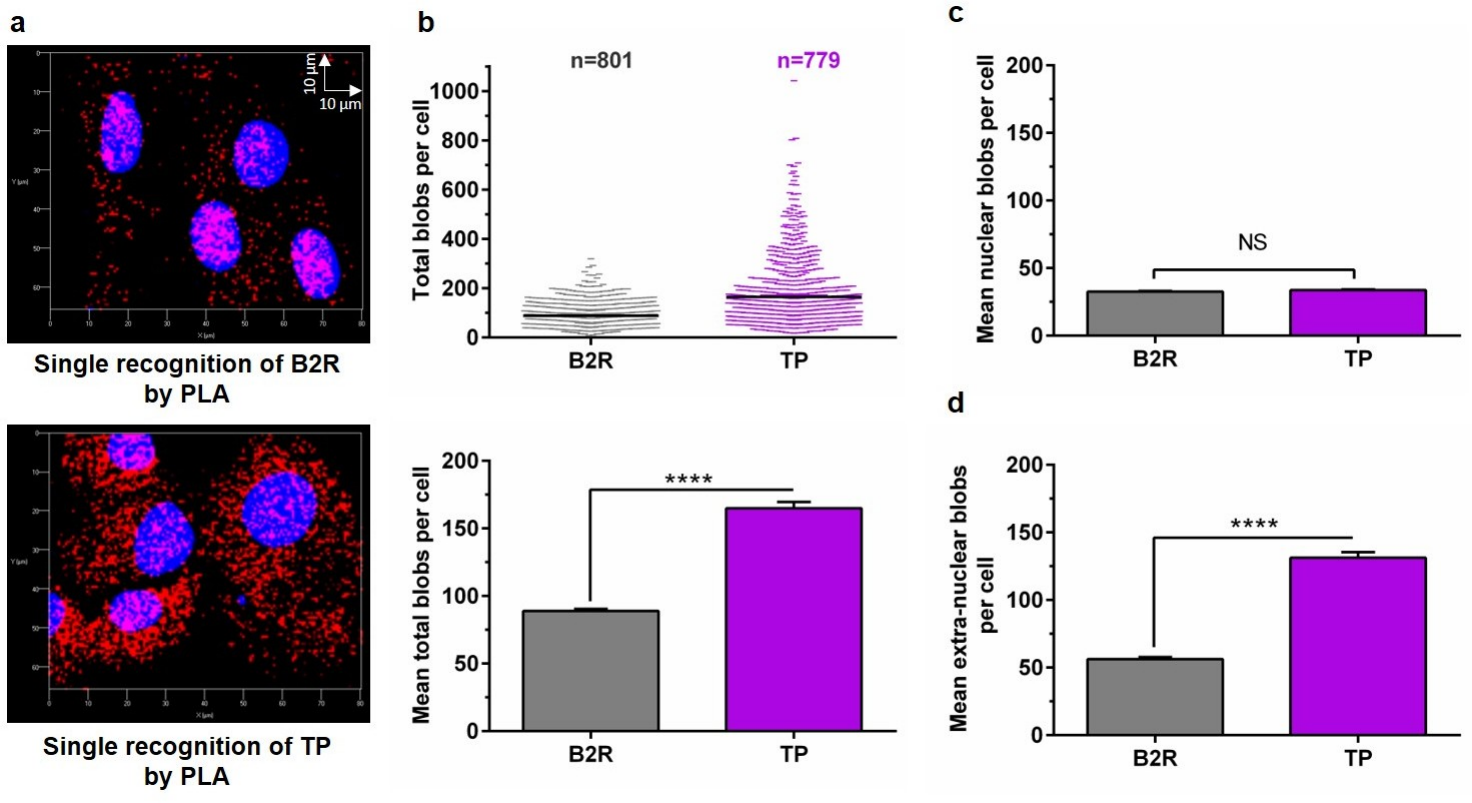
Quantitative analysis of single GPCR (B2R or TP) occupancy in VSMC using PLA. (a): Representative 3D maximum intensity projection of z-stack images obtained for RASMC analyzed by PLA for single B2R or TP expression at basal conditions (red). Nuclei appear in blue. (b): Scatter plot representing single cell analysis of the total number of PLA blobs per cell for B2R (n = 801 cells) or TP (n = 779 cells). The mean total blobs per cell is also plotted for B2R versus TP. Bar graphs representing mean ± SEM of nuclear blobs (c) or extra-nuclear blobs (d) per cell were plotted for B2R or TP. Statistical analysis was conducted using Mann-Whitney Rank Sum t-test. N = 3 independent experiments. (NS): not statistically significant; (****): p ≤ 0.0001.

Using the single cell analysis feature of BlobFinder, we analyzed the z-stacks of 801 cells (for B2R occupancy) and 779 cells (for TP occupancy). The PLA signals representing B2R or TP expression in RASMC appeared as red blobs overlapping and surrounding the blue nuclei (Fig 3A). A p-value of p ≤ 0.0001 (Mann-Whitney Rank Sum t-test) was achieved for the difference in the median total number of blobs between B2R (82 blobs) and TP (125 blobs), respectively (Fig 3B). Fig 3B also displays the bar graphs representing (mean ± SEM) total blobs of B2R and TP.

Further analysis of the subcellular distribution of B2R and TP was applied. The PLA blobs representing each of B2R or TP appeared as distinct red blobs that merged at the nuclear region for either B2R or TP, indicating that a profound amount of each of these receptors is constitutively available in the nucleus at the basal level (Fig 3A). Previous evidence exists for the possible nuclear localization of each of B2R (in rat hepatocytes) [39] and TP (in oligodendrocytes) [40], however, this is the first study that documents the nuclear localization of either receptors in VSMC.

By dividing the mean total PLA blobs of B2R over that of TP, an almost 1:2 ratio of total B2R:TP was obtained. Interestingly, however, B2R and TP appeared to be evenly spread along the nuclei with a 1:1 ratio of nuclear B2R:TP, regardless of the unequal total number of receptors in the cells (Fig 3C). We next calculated the percentage of nuclear blobs of each of B2R or TP versus the extranuclear ones, and the difference between median percentages of nuclear B2R (40.57%) versus nuclear TP (24.29%) blobs was significantly different, with a p-value ≤ 0.0001; Mann-Whitney Rank Sum t-test (S2B Fig).

#### b- Investigation of the effect of single and double ligand stimulation on B2R-TP proximity and subcellular localization in RASMC

We next investigated the likelihood of B2R-TP proximity and subcellular localization in the presence or absence of agonist stimulation in RASMC by in-situ PLA. We were interested in not only identifying whether B2R and TP colocalized at the basal level, but also in deciphering the agonist-modulatory influences on B2R-TP proximity and localization. Given that the co-stimulation of RASMC with subthreshold BK plus IBOP resulted in substantially increased ERK1/2 activation versus single stimulation with IBOP alone, we were interested in determining whether dual agonist stimulation of B2R and TP would affect their heteromerization frequency, and possibly their localization.

**Fig 4:**
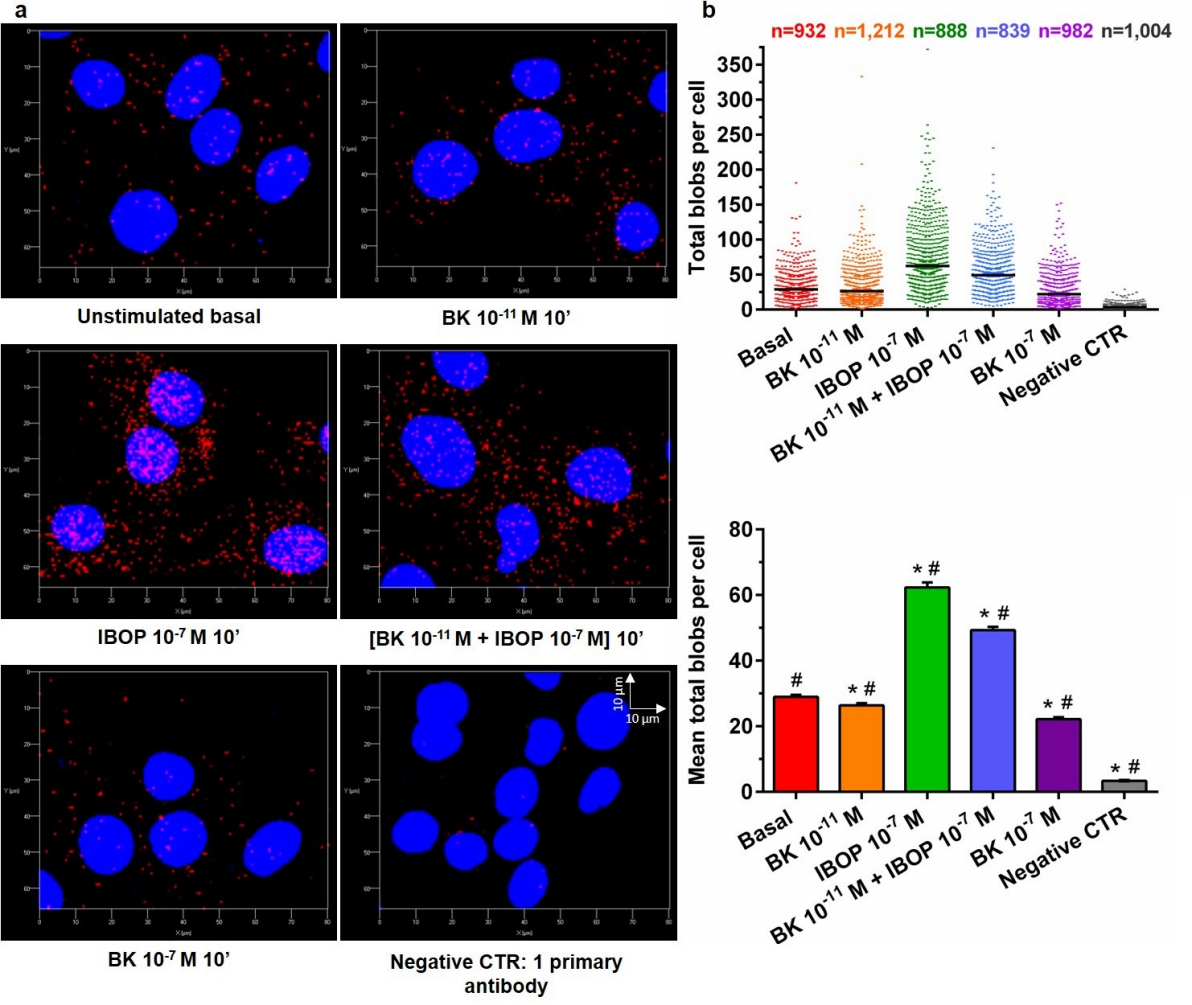
Quantitative analysis of B2R-TP proximity in VSMC using dual receptor PLA recognition workflow. (a): Representative 3D images of B2R-TP PLA blobs (red) obtained for RASMC that were either kept untreated, or stimulated with BK, IBOP, or combinations for 10 min. (b): Scattergram of the total number of PLA blobs per cell for B2R-TP heteromer. The total number of cells analyzed is shown at the top of the scattergram for each treatment group. Bar graphs of the mean± SEM total B2R-TP PLA blobs per cell were also plotted. Statistical analyses were performed using Kruskal-Wallis One Way ANOVA (p ≤ 0.0001) followed by Dunn’s multiple comparison analysis. N = 3 independent experiments. (*) p < 0.05, versus basal; (#): p < 0.05, all pairwise comparisons.

We thus utilized the dual recognition PLA workflow to quantify B2R-TP interactions at the subcellular level per cell in a minimum of 839 cells per treatment condition. Single cell PLA signals representing B2R-TP were quantified in RASMC that were either kept untreated (red) or were stimulated with BK 10^−11^ M (orange), IBOP 10^−7^ M (green), [BK 10^−11^ M + IBOP 10^−7^ M] (blue), or BK 10^−7^ M (purple) for 10 min (Fig 4B). The treatment duration of 10 min promotes treatment-dependent modifications in B2R and TP activity without inducing a change in their protein expression and matches the peak time of ERK1/2 phosphorylation as obtained in our optimization for ERK1/2 profiling analysis (S1B and S1D Fig).

The B2R-TP PLA signals appeared as red puncta overlapping and adjoining the blue nuclei in the 3D re-constructed images, (Fig 4A). As expected, constitutive B2R-TP complexes were detected in unstimulated RASMC with a (mean ± SEM) total blob count of (28.98 ± 0.62) blobs per cell. However, a profound increase in B2R-TP blob counts was seen in IBOP- and (BK 10^−11^ M + IBOP 10^−7^ M)-treated RASMC, with the former inducing a maximum increase in B2R-TP complexes to about double that of basal. Single stimulation with IBOP substantially increased the (mean ± SEM) total blob count to (62.33 ± 1.51) blobs per cell. Likewise, the increase in (mean ± SEM) total B2R-TP blob count was also very significant (49.28 ± 1.04) following dual stimulation of the receptors with (BK 10^−11^ M + IBOP 10^−7^ M). On the other hand, single stimulation of RASMC with subthreshold concentration (10^−11^ M) or otherwise effective concentration (10^−7^ M) of BK resulted in slightly lower detected blobs per cell, (26.38 ± 0.67) and (22.15 ± 0.64), respectively, as compared to the unstimulated control. All pairwise comparisons against the unstimulated control, or between groups reflected statistically significant differences with p < 0.05 (Fig 4B).

Further assessment of the subcellular prevalence of B2R-TP signals in agonist-stimulated RASMC shows that the nuclear versus extranuclear dispersion pattern was still detected in cells from all treatment groups (Fig 4A). However, except for the insignificant difference in median nuclear blobs in BK 10^−11^ M-treated versus unstimulated RASMC, all pairwise comparisons against the unstimulated control, or between groups demonstrated statistically significant differences in the median nuclear blobs with a p < 0.05. Interestingly, knowing that IBOP is of lipid formulation and thus could possibly cross the plasma membrane, IBOP-stimulated cells, and, to a lesser extent, (BK plus IBOP)-stimulated RASMC also revealed a parallel increase in B2R-TP nuclear interactions as compared to the constitutively available nuclear B2R-TP complexes. IBOP-stimulated cells showed the highest nuclear count of (18.22 ± 0.51) B2R-TP blobs followed by (12.58 ± 0.28) for (BK plus IBOP) co-stimulated cells, as compared to the unstimulated controls (7.06 ± 0.15); (S2C Fig).

### Assessment of B2R-TP interaction by co-IP

**Fig 5:**
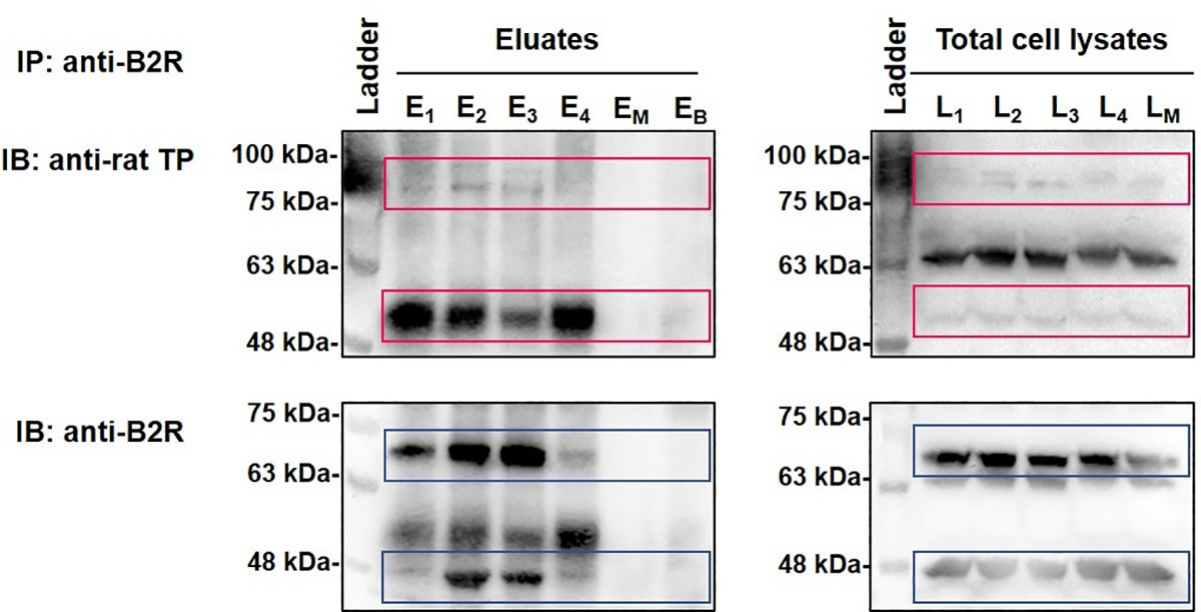
B2R-TP interactions in RASMC as revealed by co-IP followed by SDS PAGE. RASMC lysates were immunoprecipitated with anti-B2R followed by immunoblotting with anti-TP (upper panels) and anti-B2R (lower panels) antibodies, successively. E and L represent eluates and matching lysates of unstimulated RASMC (E1, L1), or RASMC that were stimulated with BK 10−11 M (E2, L2), IBOP 10−7 M (E3, L3), or [BK 10−11 M + IBOP 10−7 M] (E4, L4) for 10 min. EM and LM represent eluates and matching lysates of mock co-IP condition. (EB) represents eluates of a control co-IP condition, whereby Dynabeads-protein-A-anti-B2R were incubated with PBS instead of RASMC lysates. Images are representative of three qualitatively similar independent experiments. A denatured broad molecular weight protein ladder was loaded in parallel (upper and lower left-hand lanes).

The PLA data revealed a constitutive and agonist-modulated proximity between B2R and TP in RASMC suggestive of a likelihood of B2R-TP heteromerization. Thus, we next utilized the co-IP workflow followed by SDS PAGE to investigate the likelihood of B2R-TP interactions in RASMC. Dynabeads-protein-A were crosslinked to anti-B2R to avoid its later co-elution with target protein complexes prior to incubation with protein lysates of RASMC. E and L represent eluates and matching lysates of RASMCs that were either kept untreated (E_1_, L_1_), or were stimulated with BK 10^−11^ M (E_2_, L_2_), IBOP 10^−7^ M (E_3_, L_3_), or [BK 10^−11^ M + IBOP 10^−7^ M] (E_4_, L_4_) for 10 min. As a mock condition, the same crosslinking procedure was run in parallel in the absence of anti-B2R, and the beads were incubated with RASMC protein lysates (E_M_, L_M_). Because the molecular weight of TP monomers is about 55 kDa, which might overlap with the molecular weight of the IgG heavy chain (around 50 kDa), an additional control condition was added whereby Dynabeads-protein-A-anti-B2R were incubated with PBS instead of RASMC lysates, and the eluates (E_B_) were collected. Afterwards, the eluted immunoprecipitation complexes were loaded along with their matching total cell lysates onto SDS PAGE gel and immunoblotted for the expression of TP and B2R using anti-TP (Fig 5; upper panel) and anti-B2R (Fig 5; lower panel) antibodies, successively.

Both in the eluates and matching total cell lysates, TP-specific bands were present at 55–60 kDa, in untreated and agonist-stimulated RASMC, but were absent in the eluates (E_M_) of the mock-IP condition or the control condition (E_B_) (Fig 5; upper panel). This is in accordance with previously reported bands for TP (55 kDa) [40]. To rule out whether the heavy chain of the crosslinked anti-B2R was co-eluted with the immunoprecipitated complexes, eluates from the control condition (E_B_) were loaded onto the SDS-PAGE gel to test the efficiency of crosslinking. Visual inspection of the lane corresponding to (E_B_) at the 50–55 kDa position does not show any band in this negative control lane, indicating that our protocol was highly efficient in terms of crosslinking and elution, and that the bands realized in our experimental conditions are TP-specific. Furthermore, RASMC contained receptor bands for rat TP at 80–100 kDa, which appeared as thick smears in the eluates, and which might represent a detergent-resistant receptor homo- or hetero-dimer or a monomer interacting with another protein (Fig 5; upper panel). An additional 65 kDa band appeared only in the lanes corresponding to total cell lysates for rat TP, possibly representing a part of the TP receptor pool that is not involved in dimerization.

On the other hand, two main bands could be detected for B2R (Fig 5; lower panel), at 48 kDa and 70 kDa, in the lanes corresponding to our experimental conditions in the eluates as well as total cell lysates. The absence of these bands from the (E_M_) or (E_B_) lanes indicate that they are B2R-specific.The expected molecular weight of rat B2R is 38–40 kDa, however the primary receptor band that was enriched in the eluates of RASMC was at 48 kDa. Comparable to what was seen for TP, the ~70 kDa band seen for B2R could be a homo- or hetero-dimer that was detergent-resistant. The ~70 kDa band has been repeatedly reported for B2R in the literature [41,42].

## Discussion

Variations in plasma-dependent microenvironments following vascular injury promote phenotypic changes in VSMC that could be either acute or chronic, and that encompass changes in their morphology, proliferation, migration, protein expression, and extracellular matrix synthesis to repair the vessel wall [43]. BK and TX-A_2_ are two prominent vasoactive mediators within this microenvironment that could easily access their VSMC surface receptors following vascular injury and elicit their actions. Thus, unveiling any type of crosstalk between both systems in VSMC might help uncover novel targets for interventional strategies that tackle vascular diseases. Knowing that each of B2R and TP could form functional homo- and hetero-dimers that would affect the signaling and trafficking properties of individual GPCRs [19,44,45], we suspected a possible similar mode of interaction to occur between B2R and TP in VSMC.

Evidence supporting the heteromerization of B2R with other GPCRs has been documented in many reports. While some heteromers were constitutive, others were agonist- modulated. However, in most cases, differential signaling modulation and/or internalization properties were attributed to the heteromer as compared to individual receptors. For instance, studies conducted by AbdAlla et al., 2000, described an angiotensin AT_1_-B2R heterodimer in both A10 VSMCs and HEK293-transfected cells [46]. The formation of this complex was agonist-independent; however, it led to enhanced angiotensin II responses but decreased potency and efficacy of those of BK. Moreover, this heteromerization shifted the mode of receptor trafficking from dynamin-I- independent internalization for the individual receptors to dynamin-I- dependent sequestration of the complex. However, this seems controversial, as in a subsequent study investigating AT_1_R-B2R heteromerization in COS-7, HEK293, and NIH3T3 cells, researchers could not detect any constitutive dimerization between AT_1_R and B2R nor signaling modulation [47]. On the other hand, B2R could form spontaneous heteromers with the kinin B1R subtype when co-expressed in HEK293 cells undergoing persistent insult resulting in enhanced agonist-dependent and -independent signaling of the heteromer and the conversion of the kinin signal from B2R to B1R type [19]. B2R could also form a functional heterodimer with angiotensin AT_2_ receptor, which results in enhanced angiotensin signaling through Gα and subsequent nitric oxide production [48]. Interestingly, B2R heteromerization with the β_2_ adrenergic receptor has been validated both in-vitro and in-vivo, with functional impact on cardiac release of the tissue plasminogen activator in the myocardium [49,50]. The coupling of B2R with P_2_Y_2_ ATP receptors has also been described, whereby the formed dimer altered the internalization, signaling, and desensitization of individual receptors [51]. B2R could also constitutively heteromerize with angiotensin Mas (1–7) receptor, MasR, and elicit unique signaling properties [41]. Recently, functional B2R heteromerization has also been documented with the dopamine D2 receptor [52] and κ-opioid receptor (KOR) [53].

Likewise, studies conducted on TP have also shown the ability of each of human TPα and TPβ to form homomers or heteromers, which would affect the trafficking and signaling properties of these receptors. For instance, TPα homodimerization has been postulated as a vital event for normal TPα-Gα_q_ activation [54]. This has been supported by another report that showed that the characterization of a TPα homodimer deficient mutant (DDM) showed significant impairment in its response to agonists. Notably, this seems to have pathological implications in-vivo, since some of the mutated residues in the DDM match with two single loss-of-function TPα variants, in two recently identified patients affected by bleeding disorders [55]. On the other hand, Laroche and coworkers demonstrated that TPα, which does not internalize individually even after ligand binding, could co-internalize with TPβ secondary to the formation of a TPα-TPβ heterodimer [44]. This led to reduction in the availability of TP subtypes on the plasma membrane, and thus reduction in TP-mediated responses and signaling [56]. TPα-TPβ heteromerization might also result in conformational changes in ligand binding sites, as suggested by Wilson et al., 2007 [37]. Another example of TPα heteromerization is that reported with the prostacyclin receptor (IP) that results in enhanced TP-mediated cAMP formation [45], IP-mediated internalization of TPα [20], and relocation of TP to lipid rafts [57]. Additionally, a functional heteromer has also been described for TPα with adenosine A(1) receptors as having distinct ligand-modulated signaling [58].In-situ PLA is a microscopy-based technique that allows single cell analysis, subcellular localization, and quantification of transient and weak protein-protein interactions in-situ owing to its exceptional sensitivity, specificity, and single-molecule precision [36,59]. Based on the manufacturer’s recently updated troubleshooting guide, PLA permits the detection of proximity between two target proteins if they exist within a maximum theoretical distance of 40 nm and can theoretically detect epitopes that are within zero nm distance. Indeed, some recently published studies have utilized PLA for GPCR heteromerization detection [41,60]. In this manuscript, we used PLA to perform comprehensive analysis and quantification of the distribution of B2R and TP as well as B2R-TP interactions in native VSMC as they exist within their physiological context.

Quantitative single cell analysis of rat B2R-TP in VSMC by PLA is suggestive of both constitutive and ligand-regulated heteromerization, whereby the quantification of B2R-TP proximity became substantial upon single stimulation with IBOP or co-stimulation with subthreshold BK plus IBOP. It was interesting to find constitutive and ligand-modulated nuclear B2R-TP complexes in rat VSMC, which require further investigation for their possible signaling functionality.

While evidence for nuclear B2R [39] and TP [40] has been previously documented, this is the first study that shows nuclear B2R and TP localization in VSMC. It has been previously suggested that the nuclear import of B2R could be facilitated by its nuclear localization sequence [61]. Although it is yet unclear how some GPCR ligands promote their co-internalization with their cognate receptors to the nuclear membranes, this feature has been documented for several GPCRs especially peptide GPCRs such as that of the PAR2 and PAF [62,63]. On the other hand, lipid-based ligands, such as those of the prostaglandin GPCRs, example PGE2, might also be made in-situ via localized biosynthetic machinery on the nuclear membrane, and easily diffuse through the lipid bilayer [64]. In our study, since IBOP is a bioactive lipid that is more stable than the prostaglandin, TXA_2_, one possible mechanism might involve the free diffusion of IBOP through the plasma membrane to reach its nuclear receptors.

**Fig 6:**
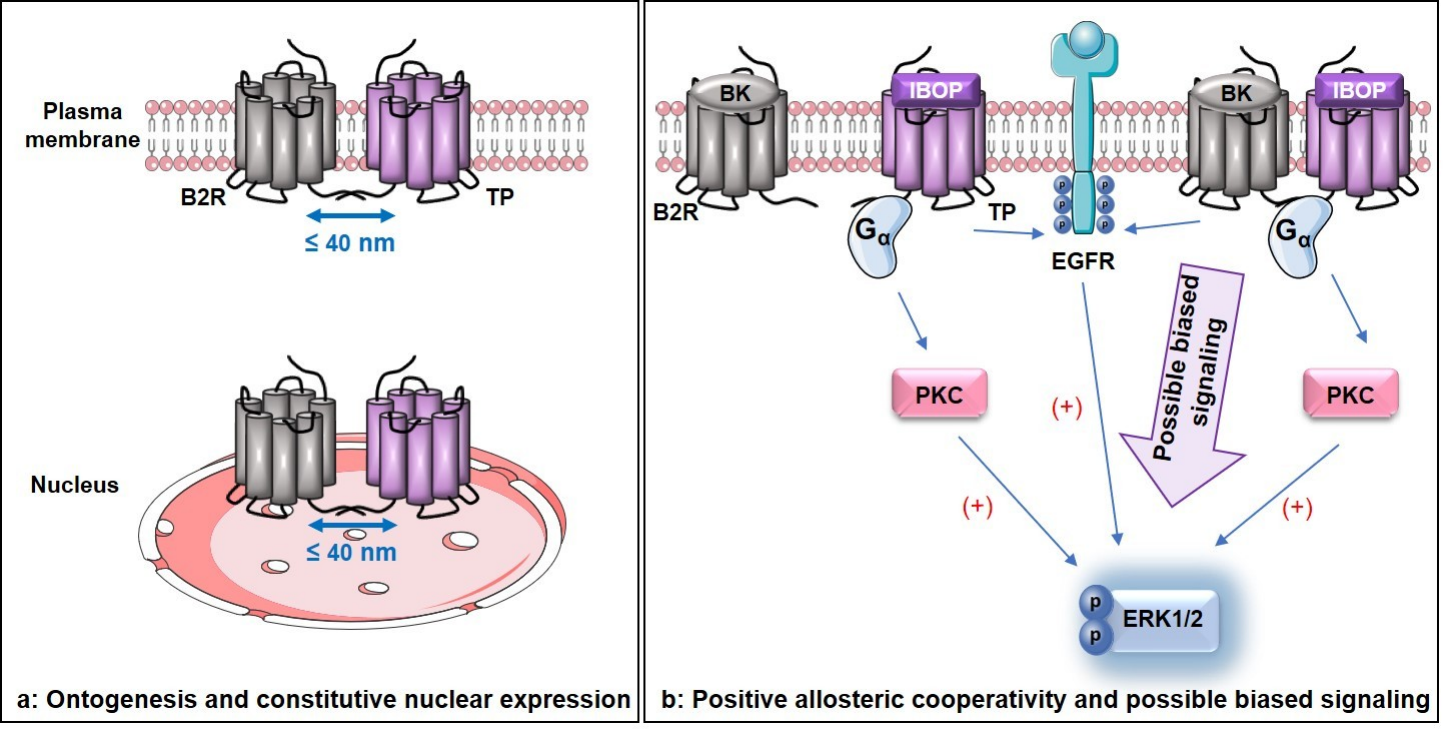
The unique aspects of B2R-TP crosstalk in VSMC. (a): Constitutive B2R-TP interaction exists at the plasma membrane and nuclei implying possible B2R-TP association early during ontogenesis. (b): BK provokes positive allosteric modulation on IBOP resulting in synergistic ERK1/2 signaling possibly mediated via a biased signaling pathway in rat VSMC.

Constitutive proximity between B2R and TP could be visualized at several subcellular compartments in rat VSMC, both nuclear and extra-nuclear, implicating their possible association during ontogenesis (Fig 6A). The first stage in a GPCR life cycle includes its ontogenesis, which entails receptor synthesis, quality control in the rough endoplasmic reticulum, maturation in the Golgi, and translocation to the plasma membrane [65,66]. At early stages of GPCR biosynthesis, dimerization might be needed as a prerequisite for proper protein folding and maturation [67–70]. Two possible mechanisms could be leading to constitutive B2R-TP heteromerization. (a)- The individual receptors could be synthesized, properly folded, and translocated to the cell surface or nuclei as monomers, and there they get associated together. Or, (b)- the association between B2R and TP occurs during the early stages of ontogenesis, and both B2R and TP translocate to the nuclei or cell surface after they dimerize in the rough endoplasmic reticulum or Golgi. Additional experiments are essential to decipher the exact mechanism of constitutive B2R-TP association in VSMC. Nonetheless, the 1:1 ratio of nuclear B2R:TP, regardless of the unequal total number of these receptors in VSMC, behooves us to question whether these cells tend to keep a uniform constitutive repertoire of nuclear GPCRs.

Complementary to the results of the PLA technique, data from the co-IP experiments showed that bands corresponding to each of B2R and TP could be detected within the eluted protein complexes, following the immunoprecipitation of RASMC protein lysates with anti-B2R. This implies that B2R and TP could be closely associated in RASMC. The eluted B2R-TP complexes were seen at basal levels, as well as following treatment with single agonists (BK or IBOP) alone, or in combination. Thus, findings from the PLA and co-IP experiments support the likelihood of B2R-TP heteromerization in RASMC.

In our proposed model of B2R-TP crosstalk, the functional consequences of B2R-TP association were evident in terms of ligand pharmacology and downstream signaling. At the single ligand stimulation level with the TP agonist, IBOP, in rat VSMC, IBOP-bound TP fostered functional G-protein coupled signaling mainly via a PKC-dependent ERK1/2 phosphorylation besides EGFR transactivation. Meanwhile, synergy between a “fixed” minimally effective concentration of BK (10^−11^ M) and IBOP (in a series of concentrations) was demonstrated for several binary BK-IBOP combinations resulting in several distinct folds of ERK1/2 phosphorylation. This was confirmed by the 2-fold increase in the maximal effects (E_max_) and 18-fold decrease in EC_50_ that were attained by the combination treatment as compared to treatment with IBOP alone, the data obtained from the normalized isobologram, and the data inferred from CI values and the CI-Fa plot. Moreover, dual stimulation with a subthreshold BK (10^−11^ M) and above-EC_50_ concentration of IBOP resulted in synergistic ERK1/2 activation that only partially involved PKC and EGFR, and was totally inhibited by the TP antagonist, SQ29548. One possibility could be the activation of a biased signaling pathway that could be predominantly leading to synergistic ERK1/2 activation. However, this remains elusive, as more experiments are necessary to decipher the exact mechanism of (BK plus IBOP)-induced ERK1/2 synergy in RASMC.

Two GPCRs could functionally crosstalk through multiple ways, important among which are receptor heteromerization, modulation of scaffold and kinase proteins, and regulation of receptor gene expression [71]. Thus, signaling crosstalk between both B2R and TP at the ERK1/2 pathway could be plausible, independent of receptor dimerization.

However, owing to the proximity detected between B2R and TP by PLA, and their interaction as inferred from their co-elution by co-IP, one possible interpretation for the synergistic ERK1/2 activation is that subthreshold BK might be acting as a positive allosteric modulator, that upon binding its B2R, induces conformational changes in B2R-TP complex that boost the binding of IBOP to its receptor and modulate the downstream signaling, resulting in synergistic ERK1/2 activation (Fig 6B). This mode of positive cooperativity has been documented for several GPCR heteromers. For instance, in the case of (cannabinoid CB1 receptor—δ opioid receptor) heterodimers, both in recombinant systems expressing both receptors and endogenous tissues, binding of a subthreshold dose of cannabinoid CB1 receptor agonist or a selective antagonist potentiated the binding and consequently signaling of δ opioid receptor agonist [72].

Taken together, our findings demonstrate, for the first time, the proximity and interaction between B2R and TP in VSMC that was constitutive and agonist-modulated with possible implications on downstream signaling, pointing to heteromerization fingerprints between B2R and TP in rat VSMC. However, more insights are required for establishing the molecular mechanism behind B2R-TP heteromerization and its possible functional implications on the vasculature.

## Supporting information

S1 Fig RASMC characterization and optimization for BK or IBOP-induced ERK1/2 profiling. **(a):** α-SMA expression in RASMC. (1) Representative image obtained by applying 3D maximum intensity projection on z-stacks of RASMC at passage 3 stained with mouse anti-α-SMA antibody followed by incubation with anti-mouse-Alexa 568 secondary antibody (red). Nuclei were counterstained with DAPI (blue). (2) As a negative control, no red signal was seen when cells were incubated with anti-mouse AlexaFluor568 secondary antibody in the absence of anti-α-SMA antibody. (b-d) Optimization for ERK1/2 profiling in RASMC treated with BK or IBOP. **(b):** Time- course curves of “fold/basal” ERK1/2 phosphorylation in RASMC stimulated with IBOP (violet circles) or BK (green squares). **(c):** BK induces ERK1/2 phosphorylation in a concentration- dependent manner. **(d):** Representative western blots for curves seen in **(b)** and **(c)**. Results are mean ± SEM of at least three independent experiments. *: p < 0.05 as compared to basal; One Way ANOVA.
(TIF)

S2 Fig In-situ PLA workflow in RASMC and subcellular quantification of B2R, TP, or B2R-TP PLA blobs. **(a):** Single versus dual receptor PLA recognition in RASMC using the in-situ PLA workflow. Fixed cells were incubated with rat anti-TP and mouse anti-B2R antibodies. This was followed by incubation with two PLUS and MINUS PLA probes. If both probes were within enough proximity, a continuous single stranded DNA circle was formed upon ligation by T4 DNA ligase, and the signal was further amplified by rolling circle amplification, utilizing one of the PLA probes as a primer. The amplified signal was then detected by hybridization with fluorescent detection probes (Alexa 594). Each individual fluorescent blob represents the amplified signal from one detected pair of PLA probes. Z-stacks were acquired by confocal microscopy and the blobs were quantified by the BlobFinder software for subsequent data analysis. **(b):** The percentage subcellular distribution of PLA blobs was calculated for nuclear versus extra-nuclear regions per cell for single B2R or TP occupancy and plotted. Statistical analysis was conducted using Mann-Whitney Rank Sum t-test. N = 3 independent experiments. (NS): not statistically significant; (****): p ≤ 0.0001. **(c):** Bar graphs representing mean ± SEM of nuclear B2R-TP PLA blobs per cell in treated versus untreated RASMC. Statistical analyses were performed using Kruskal-Wallis One Way ANOVA (p ≤ 0.0001) followed by Dunn’s multiple comparison analysis. N = 3 independent experiments. (*) p < 0.05, versus the unstimulated control; (#): p < 0.05, between groups. (NS): not statistically significant.
(TIF)

S1 Table Statistical analysis of IBOP and (BK+IBOP) treated RASMC. *: within same treatment group (BK+IBOP dose response curve); **#**: against another treatment group (IBOP dose response curve versus BK+IBOP dose response curve); N: number of independent experiments.
(DOCX)

S2 Table CI values and the corresponding doses of BK and IBOP used as single agents or in combination at several Fa levels for the construction of Fa-CI plot. *: combination is synergistic if CI < 1, additive if CI = 1, and antagonistic if CI > 1.
(DOCX)

S3 Table Primary antibodies and corresponding PLA probes used in PLA experiments. As the specific antibodies we used were directed against epitopes located on the intracellular domains, within the carboxy terminal tails of B2R or TP, cell permeabilization was conducted in our PLA workflow prior to incubation with respective antibodies.
(DOCX)
