# Supplementary figures and images for: Heteromerization fingerprints between bradykinin B2 and thromboxane TP receptors in native cells
Source: PLoS One. 2019 May 14;14(5):e0216908. doi: 10.1371/journal.pone.0216908 (PMC6516669; doi:10.1371/journal.pone.0216908)

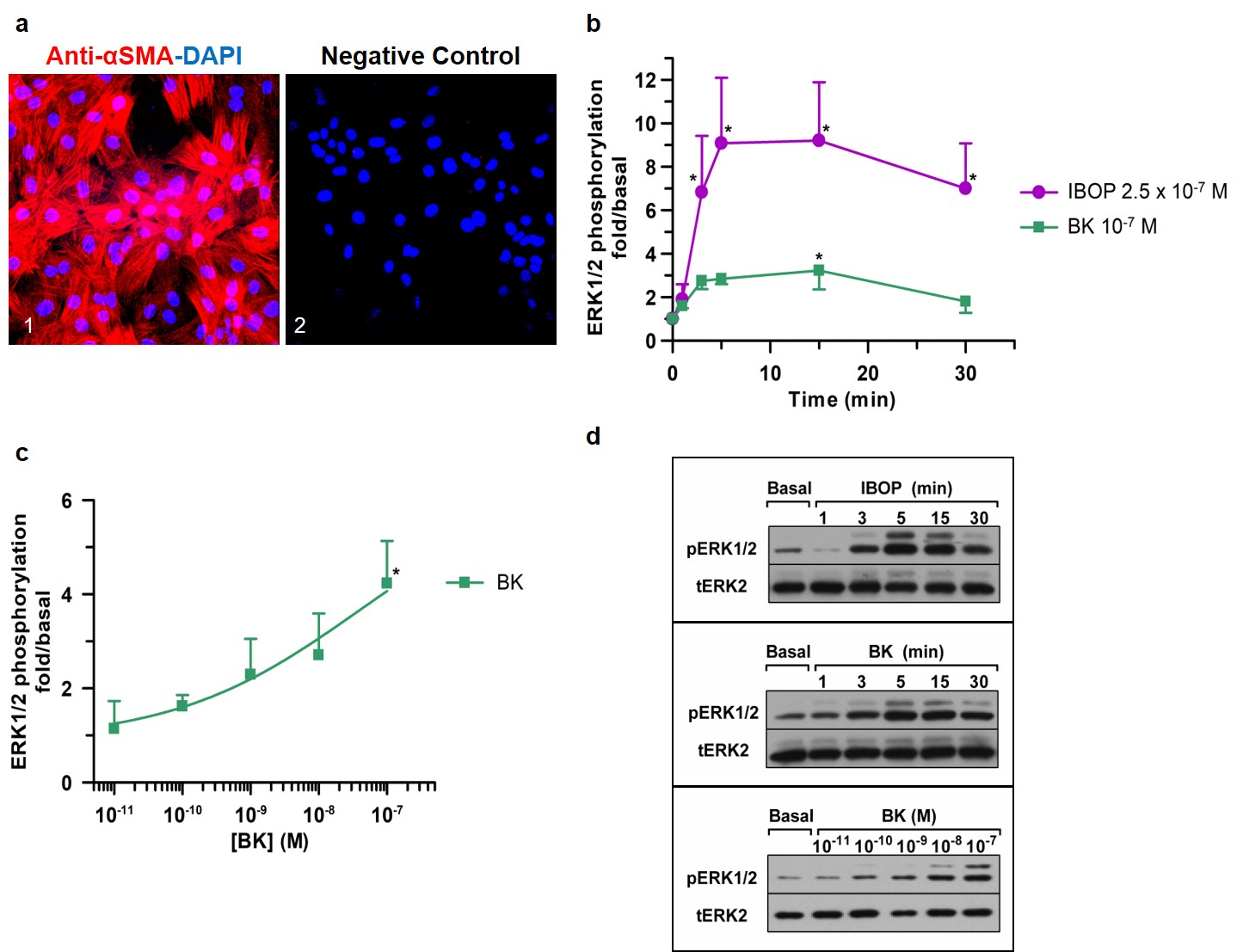

Supplement: S1 Fig — (a): α-SMA expression in RASMC. (1) Representative image obtained by applying 3D maximum intensity projection on z-stacks of RASMC at passage 3 stained with mouse anti-α-SMA antibody followed by incubation with anti-mouse-Alexa 568 secondary antibody (red). Nuclei were counterstained with DAPI (blue). (2) As a negative control, no red signal was seen when cells were incubated with anti-mouse AlexaFluor568 secondary antibody in the absence of anti-α-SMA antibody. (b-d) Optimization for ERK1/2 profiling in RASMC treated with BK or IBOP. (b): Time- course curves of “fold/basal” ERK1/2 phosphorylation in RASMC stimulated with IBOP (violet circles) or BK (green squares). (c): BK induces ERK1/2 phosphorylation in a concentration- dependent manner. (d): Representative western blots for curves seen in (b) and (c). Results are mean ± SEM of at least three independent experiments. *: p < 0.05 as compared to basal; One Way ANOVA. (TIF) [file pone.0216908.s001.tif]

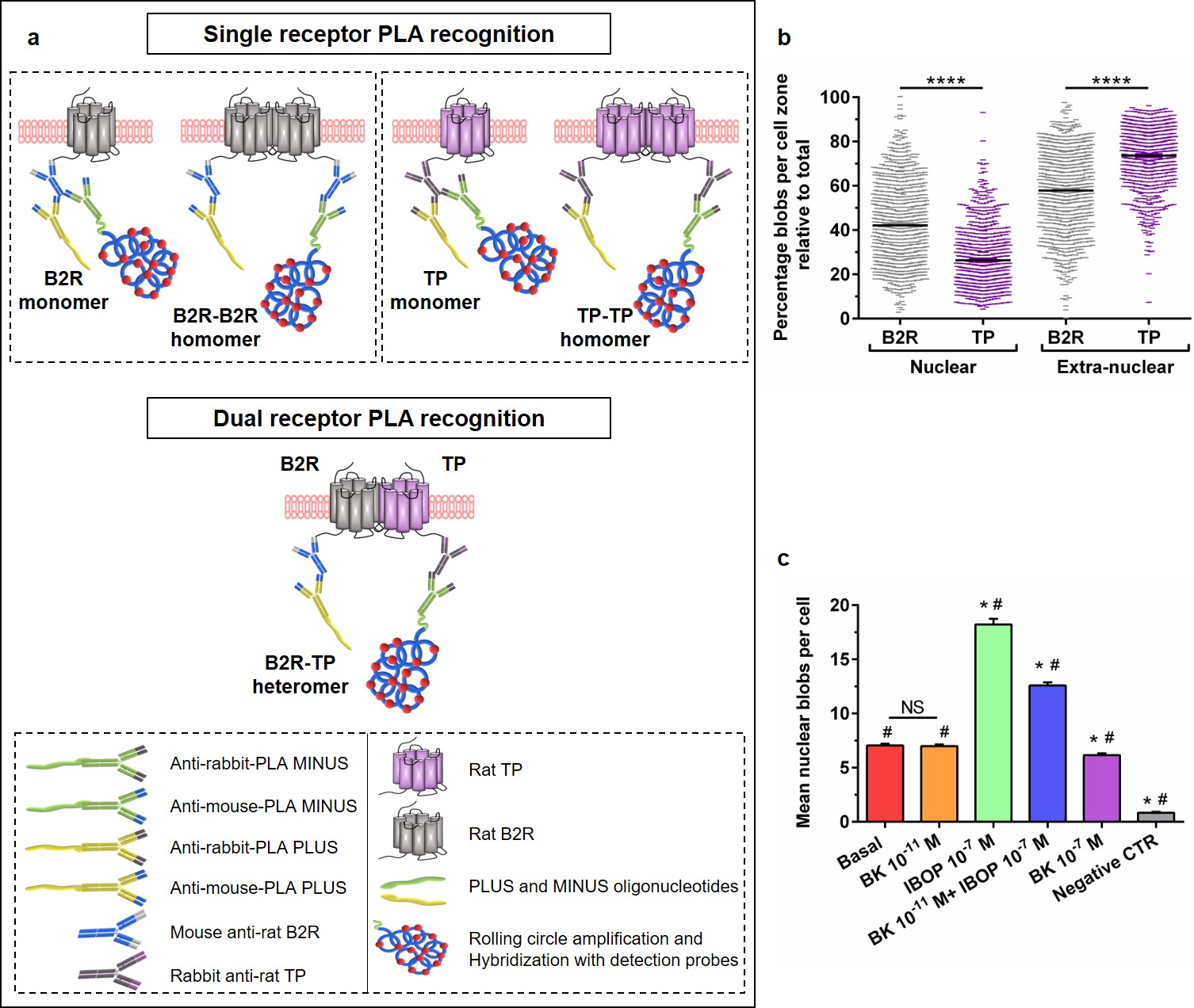

Supplement: S2 Fig — (a): Single versus dual receptor PLA recognition in RASMC using the in-situ PLA workflow. Fixed cells were incubated with rat anti-TP and mouse anti-B2R antibodies. This was followed by incubation with two PLUS and MINUS PLA probes. If both probes were within enough proximity, a continuous single stranded DNA circle was formed upon ligation by T4 DNA ligase, and the signal was further amplified by rolling circle amplification, utilizing one of the PLA probes as a primer. The amplified signal was then detected by hybridization with fluorescent detection probes (Alexa 594). Each individual fluorescent blob represents the amplified signal from one detected pair of PLA probes. Z-stacks were acquired by confocal microscopy and the blobs were quantified by the BlobFinder software for subsequent data analysis. (b): The percentage subcellular distribution of PLA blobs was calculated for nuclear versus extra-nuclear regions per cell for single B2R or TP occupancy and plotted. Statistical analysis was conducted using Mann-Whitney Rank Sum t-test. N = 3 independent experiments. (NS): not statistically significant; (****): p ≤ 0.0001. (c): Bar graphs representing mean ± SEM of nuclear B2R-TP PLA blobs per cell in treated versus untreated RASMC. Statistical analyses were performed using Kruskal-Wallis One Way ANOVA (p ≤ 0.0001) followed by Dunn’s multiple comparison analysis. N = 3 independent experiments. (*) p < 0.05, versus the unstimulated control; (#): p < 0.05, between groups. (NS): not statistically significant. (TIF) [file pone.0216908.s002.tif]
