# Supplementary material for: Heteromerization fingerprints between bradykinin B2 and thromboxane TP receptors in native cells
Source: PLoS One. 2019 May 14;14(5):e0216908. doi: 10.1371/journal.pone.0216908 (PMC6516669; doi:10.1371/journal.pone.0216908)
Supplement: S1 Table — *: within same treatment group (BK+IBOP dose response curve); #: against another treatment group (IBOP dose response curve versus BK+IBOP dose response curve); N: number of independent experiments. (DOCX) [file pone.0216908.s003.docx]

**S1 Table. Statistical analysis of IBOP and (BK+IBOP) treated RASMC.**

| **Treatment** | **Fold/basal ERK1/2 phosphorylation (mean ± SEM)** | **P-value** | **Difference of means** | **N** | **Statistical test** |
| --- | --- | --- | --- | --- | --- |
| BK 10^-11^ M  + IBOP 10^-9^ M | 11.14 ± 2.09 | ***** p = 0.0105 (vs. basal) |  | 5 | One Way ANOVA, post *hoc* Bonferroni |
| BK 10^-11^ M  + IBOP 10^-8^ M | 14.73 ± 2.96 | ***** p = 0.0005 (vs. basal) |  | 5 | One Way ANOVA, post *hoc* Bonferroni |
| BK 10^-11^ M  + IBOP 10^-7^ M | 17.75 ± 3.18 | ***** p < 0.0001 (vs. basal) |  | 5 | One Way ANOVA, post *hoc* Bonferroni |
| IBOP 10^-9^ M alone | 2.58 ± 1.24 | **#** p = 0.0007 (vs. BK 10^-11^ M + IBOP 10^-9^ M) | 8.56 | 5 | Two Way ANOVA, post *hoc* Holm-Sidak |
| IBOP 10^-8^ M alone | 5.22 ± 0.68 | **#** p = 0.0002 (vs. BK 10^-11^ M + IBOP 10^-8^ M) | 9.51 | 5 | Two Way ANOVA, post *hoc* Holm-Sidak |
| IBOP 10^-7^ M alone | 8.19 ± 2.38 | **#** p = 0.0002 (vs. BK 10^-11^ M + IBOP 10^-7^ M) | 9.56 | 5 | Two Way ANOVA, post *hoc* Holm-Sidak |

*****: within same treatment group (BK+IBOP dose response curve); **#**: against another treatment group (IBOP dose response curve versus BK+IBOP dose response curve); N: number of independent experiments
