# Supplementary material for: Heteromerization fingerprints between bradykinin B2 and thromboxane TP receptors in native cells
Source: PLoS One. 2019 May 14;14(5):e0216908. doi: 10.1371/journal.pone.0216908 (PMC6516669; doi:10.1371/journal.pone.0216908)
Supplement: S2 Table — *: combination is synergistic if CI < 1, additive if CI = 1, and antagonistic if CI > 1. (DOCX) [file pone.0216908.s004.docx]

**S2 Table. CI values and the corresponding doses of BK and IBOP used as single agents or in combination at several Fa levels for the construction of Fa-CI plot.**

| **E_i_: Effect**  **(fold/basal ERK1/2 phosphorylation)** | $\mathbf{F}\boldsymbol{=}\frac{\text{E}\text{i}}{\text{E}\text{max}\text{ }\text{(IBOP alone)}}$ | **Single treatment** | | **Combination treatment** | |  |
| --- | --- | --- | --- | --- | --- | --- |
|  |  | **IBOP (M)**  (D_x, IBOP_ ) | **BK (M)**  (D _x, BK_ ) | **IBOP (M)**  (D_IBOP_ ) | **BK (M)**  (D_BK_ ) | **CI *** |
| 2 | 0.2144 | 2.484 x10^-11^ | ---------- | 7.706 x10^-13^ | 10^-11^ | 0.0501 |
|  |  | ---------- | 5.238 x10^-10^ |  |  |  |
| 3.5 | 0.3752 | 2.859 x10^-9^ | ---------- | 1.075 x10^-11^ | 10^-11^ | 0.0041 |
|  |  | ---------- | 2.749 x10^-8^ |  |  |  |
| 4 | 0.4288 | 4.382 x10^-9^ | ---------- | 1.787 x10^-11^ | 10^-11^ | 0.0042 |
|  |  | ---------- | 8.578 x10^-8^ |  |  |  |
| 5 | 0.5360 | 8.797 x10^-9^ | ---------- | 4.056 x10^-11^ | 10^-11^ | 0.0046 |
|  |  | ---------- | 1.118 x10^-6^ |  |  |  |
| 6 | 0.6432 | 1.648 x10^-8^ | ---------- | 7.915 x10^-11^ | 10^-11^ | 0.0048 |
|  |  | ---------- | 1.214 x10^-4^ |  |  |  |

*****: combination is synergistic if CI < 1, additive if CI = 1, and antagonistic if CI > 1
