# Supplementary material for: Heteromerization fingerprints between bradykinin B2 and thromboxane TP receptors in native cells
Source: PLoS One. 2019 May 14;14(5):e0216908. doi: 10.1371/journal.pone.0216908 (PMC6516669; doi:10.1371/journal.pone.0216908)
Supplement: S3 Table — As the specific antibodies we used were directed against epitopes located on the intracellular domains, within the carboxy terminal tails of B2R or TP, cell permeabilization was conducted in our PLA workflow prior to incubation with respective antibodies. (DOCX) [file pone.0216908.s005.docx]

**S3 Table. Primary antibodies and corresponding PLA probes used in PLA experiments.**

| **PLA experiment** | **Primary antibodies** | **PLA probes** |
| --- | --- | --- |
| Dual receptor recognition (B2R-TP) | Mouse monoclonal anti-B2R BD Biosciences; cat#: 610452 (dilution 1/50) | Anti-mouse PLUS PLA probe |
|  | Rabbit polyclonal anti-rat TP (dilution 1/250) | Anti-rabbit MINUS PLA probe |
| Single receptor recognition (TP) | Rabbit polyclonal anti-rat TP (dilution 1/250) | Anti-rabbit PLUS PLA probe |
|  |  | Anti-rabbit MINUS PLA probe |
| Single receptor recognition (B2R) | Mouse monoclonal anti-B2R BD Biosciences; cat#: 610452 (dilution 1/50) | Anti-mouse PLUS PLA probe |
|  |  | Anti-mouse MINUS PLA probe |
| Negative control | Mouse monoclonal anti-B2R antibody was omitted | Anti-mouse PLUS PLA probe |
|  | Rabbit polyclonal anti-rat TP (dilution 1/250) | Anti-rabbit MINUS PLA probe |

As the specific antibodies we used were directed against epitopes located on the intracellular domains, within the carboxy terminal tails of B2R or TP, cell permeabilization was conducted in our PLA workflow prior to incubation with respective antibodies.
